# 

**Published:** 1871-05

**Authors:** 


### Semi-Annual Meeting of the Erie County Medical Society.

The Erie County Medical Society will hold its semi-annual meeting June 13th. Physicians in Erie County who are not members of this society should file a copy of their Diplomas in the County Clerk's Office, and become members. This is very important to their regular standing.

———:o:———

The Richmond and Louisville *Medical Journal* says: " Readers must prepare themselves to hear infinitely of *Cundurango.* It is a vegetable growth of Ecuador. It " cures venereal diseases, ulcers, and cancers." the pathology of these morbid conditions being notoriously identical, why should not one medicinal agent cure them all? Hon. Hamilton Fish, E. Rumrey Wing, Dr. Cæsares, etc., are (through the State Department) arranging a medicinal *coup d'etat*, and the medical world is warned in advance. Ricord, Cullurier, Hunter, etc., have been great stars in the pathological firmament, but these now wane; their cycle is ended. The medical world looks to Cæsares, Wing, and Fish, for future light and guidance!"

### Books and Pamphlets Received.

Insanity and its Treatment. Lectures on the treatment, Medical and Legal, of Insane patients. By G. Fielding Blandford, M. D. Oxon. With a summary of the laws in force in the United States, on the confinement of the Insane. By Isaac Ray, M. D. Philadelphia: 1871.

On Wasting Disease of Infants and Children. By Eustace Smith. M. D. Lond. Second American, from the second revised and enlarged English edition. Philadelphia: H. C. Lea, 1871.

The Change of Life in Health and Disease. A Practical Treatise on the Nervous and other Affections incidental to Women at the Decline of Life, By Edward John Tilt, M. D. Philadelphia: Lindsay & Blackston, 1871.

A Treatment on the Chronic Inflammation and Displacements of the Unimpregnated Uterus. By Wm. H. Byford, A.M., M.D. Second edition. Enlarged. With numerous illustrations. Philadelphia: Lindsay & Blackston, 1871.

Chemistry: General Medical and Pharmaceutical, including the Chemistry of the U. S. Pharmacopœia. By John Attfield, Ph. D., F.C.S. Philadelphia, H. C. Lea, 1871.

Obstetric Report of the Charity Hospital to the Medical Faculty of the University of Louisiana. By James Jones, Jr., M. D., Chief Obstetrical Clinic Medical Department Universsty of Louisiana. New Orleans.

The Medical and Scientific Circular and College Register, 1871.

The Medical Section of the work of N. B. Doubeveyer. By Doctor Gans. Carlsbad.

Annual Report of the Commissioners of Quarantine. Albany: 1871.

On Dacitylitis Syphilitica, with Observations on Syphilitic Lesions of the Joints. By R. W. Taylor, M. D. New York.

On the Study of Dermatology. By Louis A. Duhring, M. D. New York.

Medical Ethics and Medical Dissensions: A paper read before the Albany County Medical Society. By Charles A. Robertson, A.M., M.D. Albany.

Semi-Centennial Report of the Directors and Surgeons of the New York Eye and Ear Infirmary, for the year 1870.

Proceedings of the State Medical Association of Arkansas, at Little Rock, November, 1870.

Proceedings of the Homœopathic Medical Society of Ohio.

Uterine Catarrh frequently the Cause of Sterility. New Treatment. By H. E. Gantillon, M. D. Boston.

Hæmatoma Auris. By E. R. Hun, M. D.

The Atlantic Monthly; The Nation; N. Y. Observer; Every Saturday; Newspaper Reporter; Little's Living Age; Peterson's Musical Monthly; American Educational Monthly.

